# Multidisciplinary team diagnosis and treatment of pancreatic cancer: Current landscape and future prospects

**DOI:** 10.3389/fonc.2023.1077605

**Published:** 2023-03-15

**Authors:** Weirong Yao, Xiaoliang Chen, Bin Fan, Lin Zeng, Zhiyong Zhou, Zhifang Mao, Qinglin Shen

**Affiliations:** ^1^ Department of Oncology, Jiangxi Provincial People’s Hospital, The First Affiliated Hospital of Nanchang Medical College, Nanchang, China; ^2^ Department of Hepatobiliary Surgery, Jiangxi Provincial People’s Hospital, The First Affiliated Hospital of Nanchang Medical College, Nanchang, China; ^3^ Department of Radiology, Jiangxi Provincial People’s Hospital, The First Affiliated Hospital of Nanchang Medical College, Nanchang, China; ^4^ Institute of Clinical Medicine, Jiangxi Provincial People’s Hospital, The First Affiliated Hospital of Nanchang Medical College, Nanchang, China

**Keywords:** multidisciplinary team (MDT), pancreatic cancer, current landscape, future prospects, diagnosis and treatment

## Abstract

The pathogenesis of pancreatic cancer has not been completely clear, there is no highly sensitive and specific detection method, so early diagnosis is very difficult. Despite the rapid development of tumor diagnosis and treatment, it is difficult to break through in the short term and the overall 5-year survival rate of pancreatic cancer is less than 8%. In the face of the increasing incidence of pancreatic cancer, in addition to strengthening basic research, exploring its etiology and pathogenesis, it is urgent to optimize the existing diagnosis and treatment methods through standard multidisciplinary team (MDT), and formulate personalized treatment plan to achieve the purpose of improving the curative effect. However, there are some problems in MDT, such as insufficient understanding and enthusiasm of some doctors, failure to operate MDT according to the system, lack of good communication between domestic and foreign peers, and lack of attention in personnel training and talent echelon construction. It is expected to protect the rights and interests of doctors in the future and ensure the continuous operation of MDT. To strengthen the research on the diagnosis and treatment of pancreatic cancer, MDT can try the Internet +MDT mode to improve the efficiency of MDT.

## 1 Introduction

Pancreatic cancer is one of the most common malignant digestive system tumors, about 227,000 patients die of pancreatic cancer every year around the world (1–4). According to the latest data from the American Cancer Society, its incidence and mortality are almost equal, and the incidence is tenth in malignant tumors, the incidence is fifth in female malignant tumors, and fourth in male malignant tumors (5). In the UK, pancreatic cancer accounts for 5.6% and 5.3% of cancer-related deaths in men and women, respectively, ranking fifth (6). China is the largest developing country in the world, with the acceleration of urbanization, the changes of lifestyle and diet, and the aging and environment, the incidence of pancreatic cancer is faster than that of in developed countries, but the growth rate of pancreatic cancer is the fastest in the whole sphere. Although pancreatic cancer did not rank in the top five of cancer-related deaths in China, the proportion of pancreatic cancer-related deaths increased by 9% in the past decade, and this proportion also increased sharply (7). Therefore, pancreatic cancer has become a rigorous public health problem threatening human life and health, and has attracted more and more attention.

The rapid progress of pancreatic cancer leads to a very high mortality rate. In the past few decades, the level of diagnosis and treatment of pancreatic cancer has been significantly improved in China. Although the prognosis has improved slightly in recent years, the survival time of most patients with pancreatic cancer is less than one year, and the 5-year survival rate is still less than 8%. Pancreatic cancer has proved to be a major diagnosis and treatment problem faced by medical circles at home and abroad.

## 2 Current status of diagnosis of pancreatic cancer

With the rapid application of modern high-tech, the advancing diagnostic methods has been developed quickly, and a variety of new drugs are widely used clinically, tumor diagnosis and treatment has undergone unprecedented improvement, but the early diagnosis rate of pancreatic cancer is still disappointing (8–11). Early detection and diagnosis of pancreatic cancer is vital t for the survival and prognosis of patients (9).

### 2.1 Imaging examination

At present, a sort of imaging examinations has been used in the diagnosis of pancreatic masses. The methods featured by different advantages and limits, which could provide complementary evidence and confirmation of each other. A proper selection of imaging methods not only improve the diagnostic efficiency and accuracy, but also reduce the unnecessary cost.

B-ultrasound shows the size and scope of the tumor, lymph node metastasis, pancreaticobiliary dilatation, etc. It is known with the advantages of simple, noninvasive and low cost. Also, it is a common screening method for abdominal tumors. At the same time its performance easily affected by fat, intestinal gas, ascites and other factors. It is hardly to show the whole picture of pancreas and not suitable for the early diagnosis of pancreatic cancer (12).

CT scan not only identify the tumor, but also provide effective preoperative evaluation for the invasion of pancreatic surrounding tissue, lymph node and distant metastasis. It is a common imaging examination method in the diagnosis of pancreatic cancer. However, the sensitivity of CT may decline with the decrease of tumor diameter (13).

The spatial resolution of MRI is lower than CT in the diagnosis of pancreatic cancer, and the evaluation of tumor resectability is similar to that of CT. Diffusion weighted imaging (DWI) could identify small lesions of pancreatic cancer, but it is not able to distinguish tumor lesions from inflammatory lesions. Magnetic resonance cholangiopancreatography (MRCP) could obtain the image of pancreaticobiliary duct with contrast agent, which is mainly used to detect the dilatation or stenosis of pancreaticobiliary duct, but its application in the diagnosis of pancreatic cancer is limited (14).

Positron emission computed tomography (PET-CT), which combines functional imaging with anatomical imaging, plays an important role in the diagnosis, staging and recurrence detection of tumors. In addition, it is powerful to analyze the metabolism of the lesions, especially in the differential diagnosis of pancreatic cancer and benign lesions (especially pancreatic head cancer and mass type chronic pancreatitis) out. However, the cost of PET-CT is relatively high and limits its utility in pancreatic cancer early screening (15).

Ultrasound endoscopy (EUS), especially the fine needle biopsy technique (EUS FNA), has a unique diagnostic value compared with other imaging examinations. Because of the invasive nature of EUS FNA, it is not suitable for the first choice of detection of pancreatic cancer. In addition, ERCP is often used to drain bile by self-expanding stent. It is not supposed to be a valuable mean in early diagnosis of pancreatic cancer (16).

### 2.2 Tumor biomarkers

Carbohydrate antigen 19-9 (CA19-9) is a marker of pancreatic cancer. The sensitivity and specificity of CA19-9 in the diagnosis of pancreatic cancer are 79% ∼ 81% and 82% ∼ 90% (17), respectively. False positive results were found in patients with liver cirrhosis and gastrointestinal cancer.CA19-9 is not used in the early diagnosis of pancreatic cancer, but often applied to evaluate curative effect and detect postoperative recurrence. Carcinoembryonic antigen (CEA) is highly expressed in patients with pancreatic cancer, gastric cancer and colorectal cancer, but its diagnostic specificity for pancreatic cancer is poor (18). In addition, other tumor markers (such as CA242, CA50, CA72-4, etc.) are not commonly used in the diagnosis of pancreatic cancer because of their low sensitivity and specificity.

### 2.3 Liquid biopsy

#### 2.3.1 Circulating tumor cells

Circulating tumor cells (CTCs) fall off from primary or metastasis tumor cells of peripheral blood. CTC may have experienced epithelial mesenchymal transition (EMT), with stronger mobility and invasiveness, and it is easier to adhere to the vascular wall and penetrate into the blood circulation, which is an important reason for tumor metastasis. CTC has integrity of the tumor data, including not only DNA information, but also genome and proteome which is consistent with the source of tumor tissue

The value of CTC in the early diagnosis of tumor has been confirmed in many kinds of tumor research. In the mouse model of pancreatic cancer, Rhim et al. (19) found that EMT occurred in some pancreatic cells at the early stage of tumor development, and these cells were considered as early tumor cells. Before malignant transformation, pancreatic epithelial cells can be detected in blood samples of patients with pancreatic cystic lesions. These results suggest that appearance of CTC is earlier than tumor formation *in situ* and may be a tumor marker for early diagnosis of pancreatic cancer.

CTC specific gene expression could be considered as an alternative marker for early diagnosis of tumor. This kind of research mainly detects the expression of epithelial protein to validate the presence of CTC. For example, Soeth et al. (20) detected cytokeratin 20 (CK20) in bone marrow and venous blood of patients with pancreatic cancer, and found that high level of CK20 was associated with tumor staging of UICC. Zhang et al. (21) combined immunostaining of CK, CD45, DAPI and fluorescence *in situ* hybridization (FISH) with chromosome 8 centromere probe (CEP8) method to improve the identification efficiency of CK-/diploid CTC in pancreatic cancer.

CTC also be taken as a marker for the diagnosis of early pancreatic cancer, asymptomatic patients and patients with normal CA19-9. Xu et al. (22) used a similar method in 40 patients. When the cut-off value set at CTC ≥ 2/7.5ml and CA19-9 > 37 μmol/L, the diagnostic rate of pancreatic cancer reached 97%. In addition, DCLK1, another marker of CTC, may also be used in the early diagnosis of pancreatic cancer. Qu et al. (23) found that the level of DCLK1 increased in patients with TNM stage I and II, but decreased in patients with TNM stage III and IV. Although CTC has great potential value in the early diagnosis of pancreatic cancer, it is difficult to capture CTCs from the blood due to the scarcity of CTCs, which limits its clinical application.

#### 2.3.2 Circulating tumor DNA

In 1977, Leon et al. found circulating tumor DNA (ctDNA) in the serum of tumor patients. In 1983, Shapiro et al. (24) first detected ctDNA in the blood of patients with pancreatic cancer. Studies have shown that ctDNA mainly comes from necrotic tumor cells, apoptotic tumor cells, CTC and exosomes secreted by tumor cells.

The length of ctDNA is about 134-144bp and the half-life is about 2 hours. It can be detected in blood, saliva, urine and other body fluids. ctDNA contains gene information of tumor cell with specific mutations. By capturing and sequencing these important DNA fragments, we could obtain tumor specific mutations information, which is helpful in tumor diagnosis and individual medication guidance.

Studies have shown that more than 90% of patients with pancreatic intraepithelial neoplasia have KRAS gene mutation, and the mutation rate of KRAS gene is directly related to the grade of pancreatic intraepithelial neoplasia (25). Detection of KRAS mutation in ctDNA is expected to be applied to the early diagnosis of pancreatic cancer. Bettegowda et al. (26) detected ctDNA in serum of 640 patients with different types and stages of tumor by using dPCR, including 155 patients with pancreatic cancer. The results revealed that the detection rate of ctDNA in patients with localized pancreatic cancer was 48%. The ratio increased with the increase of tumor clinical stage. Similarly, Sausen et al. (27) found that 43% patients being identified of ctDNA in total resectable pancreatic cancer cases. However, other studies have reported that patients with chronic pancreatitis (10% - 15%) will also have KRAS mutations, combined detection of KRAS mutations and serum creatinine levels

CA19-9 can improve the sensitivity (98%) and specificity in the diagnosis of pancreatic cancer degree (77%) (28). In addition, the study found that the methylation analysis of ctDNA can works as a potential marker of pancreatic cancer to distinguish chronic pancreatitis from pancreatic cancer (29). Although ctDNA provides a possibility for the early diagnosis of pancreatic cancer, the sensitivity of existing technologies is not satisfying, and the standardization of detection methods still needs to be settled.

#### 2.3.3 Exosomes

Exosomes are largely secreted in the process of carcinogenesis, which is different from ctDNA that released by tumor necrosis cells, exosomes are secreted by living cells, so exosomes could be distinguished earlier in the blood, which is more suitable for the early diagnosis of pancreatic cancer. Serum exosome derived proteins or miRNAs may be proper candidate markers, such as protein markers (CD44v6, TSPAN8, EpCAM, CD104) and miRNAs (miR-1246, miR-4644, miR-3976, miR-4306). The expression of these proteins and miRNAs in serum exosomes of patients with pancreatic cancer was significantly up-regulated. Combined detection of these proteins and miRNAs would effectively improve the sensitivity of diagnosis of pancreatic cancer (30). In addition, studies have shown that exosome derived DNA mutations (such as KRAS and TP53) can also be selected in the diagnosis of pancreatic cancer, and the diagnostic efficiency is better than CTC, but exosome KRAS mutations can also occur in healthy people (29). Studies have shown that GPC-1, an exosome membrane protein, can be chooses to differentiate pancreatic cancer patients from chronic pancreatitis patients and healthy people with specificity and sensitivity up to 100% (31).All the above results indicate that exosomes are expected to become a new type of biomarker. The ideal marker for early diagnosis of pancreatic cancer still supposed to be validated by a large number of studies.

Although pathological diagnosis is the gold standard for the diagnosis of pancreatic cancer, imaging diagnosis plays an important role in screening, differential diagnosis and staging of pancreatic cancer. Decisions about diagnostic management and resectablity should involve multidisciplinary consultation at a high-volume center with application of appropriate imaging studies. At present, ultrasound, Computed Tomography (CT), Magnetic Resonance Cholangiopancreatography (MRCP) and Endoscopic Ultrasonography (EUS) are the main early screening methods for pancreatic cancer. Ultrasound examination is the most economical and noninvasive examination method, and it is the first-line screening method for patients with suspected pancreatic cancer (10). However, ultrasound examination highly depends on the experience and physical condition of ultrasound doctors (32). Enhanced CT is the first choice of pancreatic imaging in the world, and it is also the first choice of postoperative evaluation of pancreatic cancer recurrence. However, enhanced CT has some radiation, which limits it as a routine screening for asymptomatic high-risk population. Endoscopic ultrasonography and cholangiopancreatography are better than CT in the early screening of pancreatic cancer (33, 34). Therefore, most scholars suggest that MRCP, Magnetic resonance imaging (MRI) and EUS should be included in the initial screening of pancreatic cancer, while CT and ERCP are excluded (9, 35). However, combined with the actual economic situation of our country, MRI examination is still carried out after ultrasound and CT examination. In addition, EUS still cannot be popularized in domestic hospitals while only installed in some large medical institutions. Although positron emission tomography/computed tomography (PET/CT) has been widely used in the diagnosis of tumors, its conventional tracer 18F-fluorodeoxyglucose (18F-FDG) has little effect in the detection of early pancreatic ductal adenocarcinoma (36, 37).

## 3 Current status of treatment of pancreatic cancer

### 3.1 Surgery

Surgical treatment is the basic treatment for pancreatic cancer, and it is also the only way to achieve the curative effect of pancreatic cancer (10, 38, 39). Recent studies have shown that less than 20.0% of pancreatic cancer patients have access to surgical treatment (40). Even after R0 resection, some patients still have postoperative tumor recurrence and distant metastasis, which affect the postoperative survival rate. For patients with unresectable pancreatic cancer, preoperative neoadjuvant therapy can be managed to transform them into resectable patients. Systemic therapy is accepted in all stages of pancreatic cancer. This includes neoadjuvant therapy (resectable or borderline resectable), adjuvant therapy, and first-line or subsequent therapy for locally advanced, metastatic, and recurrent disease (41).

#### 3.1.1 Pancreaticoduodenectomy

Pancreaticoduodenectomy (PD) was put forward by Whipple in 1935, which was also the classic surgical method for pancreatic cancer. It is mainly used for the head and neck of the pancreas (head, neck, and hook). Foreign statistics show that the most common complications of this operation include delayed gastric emptying, pancreatic fistula and wound infection incidence rate is 42%~47% (42). Bassi and other (43)studies that compared PD among different conditions, PD has no statistical significance in the proportion of complications, mortality and length of hospital stay, but the incidence of bile leakage and ascites in PD group is higher than that in pancreaticogastrostomy group, which may be due to the fact that PD group will not be invaded by pancreatic fistula, whether PD or pancreaticogastrostomy is still controversial.

#### 3.1.2 Pylorus preserving duodenectomy

Pylorus preserving pancreaticoduodenectomy (PPPD) was first proposed by Watson in 1944. It is believed that PPPD can reduce the incidence of dumping syndrome, reduce intraoperative bleeding and shorten the operation time. However, some scholars doubt that PPPD will increase the proportion of delayed gastric emptying, compared with PD, surgery does not significantly change the mortality or survival rate of patients, and does not conform to the relevant procedures of tumor resection. Therefore, the choice of surgery on PD or PPPD is still controversial.

There are many other surgical conduction, such as distal pancreatectomy, extended resection, portal vein resection, arterial resection and reconstruction, and extended lymphadenectomy (44), which have also been accepted in clinical utility.

#### 3.1.3 Minimally invasive treatment of pancreatic tumors

Due to the deep anatomic location and complex surrounding tissue structure of the pancreas, the development of minimally invasive surgery of the pancreas is more obvious than that of other digestive system tumors. With the in-depth study of minimally invasive treatment of pancreatic tumors, certain progress has been made recently. Pryor et al. (45)have studied that laparotomy and laparoscopy are the most effective methods for the treatment of pancreatic tumors. Compared with patients on different surgical treatment, the incidence of complications was 43% vs 7%, and the mortality was 29% vs 0%, which showed the obvious advantages of laparoscopic surgery compared with traditional open surgery.

With the development of medical technology, surgical robots have gradually entered people’s field of vision. Robotic surgery improves the efficiency and accuracy of surgery. Of course, there are also some disadvantages, such as the robot does not have the touch of traditional surgery, there are errors in tactile judgment. At present, the development direction of surgery is gradually toward precision and minimally invasive, which requires us to better use endoscopic technology and surgical robot, as well as the combination of the both. Regarding some experts worried that minimally invasive treatment cannot reach the R0 margin affect the OS, disease-free survival (DFS), etc., Halit et al (46) reported a study of 396 patients with borderline resectable and resectable pancreatic adenocarcinoma, minimally invasive pancreatic surgery (MIPS) was associated with better OS and DFS than open pancreatic surgery (OPS). Centralization of MIPS should be stimulated, and pancreatic surgeons should be encouraged to pass the learning curve before implementing MIPS for pancreatic adenocarcinoma in daily clinical practice.

### 3.2 Chemotherapy

Advanced patients or patients pre- and post-operative should be treated with chemotherapy (47). Pancreatic cancer is not sensitive to chemotherapy. Gemcitabine, albumin paclitaxel, fluorouracil (including capecitabine, S1) and other single drug regimens can be exerted for 6 months. Patients in good condition could be considered the combination with chemotherapy (48).

Almost all pancreatic cancer patients need chemotherapy. Early patients need postoperative chemotherapy to prevent recurrence. In late stage, chemotherapy is needed to relieve symptoms and prolong survival. Therefore, chemotherapy has always been a hot topic in the treatment of pancreatic cancer.

#### 3.2.1 Fluorouracil single therapy

Since 1950s, 5-fluorouracil (5-fluorouracil, 5-Fu) based chemotherapy has been a major chemotherapy regimen for pancreatic cancer. Although the combination of adriamycin, mitomycin C, cyclophosphamide, methotrexate vincristine and cisplatin can improve the effect of 5-FU, none of them extend the OS of patients.

#### 3.2.2 Gemcitabine single therapy

Gemcitabine (GEM) is the first chemotherapy drug that can prolong the survival period of patients with pancreatic cancer. In a randomized controlled trial (49), 126 patients with advanced pancreatic cancer were divided into two groups. One group received GEM treatment and the other group received 5-Fu treatment. The clinical benefits of the two groups were evaluated by pain index, Karnofsky (KPS) and body mass. The results showed that GEMC group had better clinical benefits (23.8% vs 4.2%, P = 0.0022); At the same time, the mOS of GEM group was longer than that of 5-FU group (5.65mo vs 4.41mo, p=0.0025), and the one-year survival rate was higher than that of 5-FU group (18% vs 2%, P = 0.0025). Therefore, GEM is classified as a first-line chemotherapeutic agent for advanced pancreatic cancer.

#### 3.2.3 GEM based combination chemotherapy

After the single efficacy of GEM was verified, a series of GEM based combination chemotherapy developed rapidly from the 1990s to the early 21st century. The efficacy of GEM combined with capecitabine was verified in two clinical phase III trials. Cunningham et al. (50) selected 533 patients with advanced pancreatic cancer were randomly divided into two groups, one group received chemotherapy combined with GEM plus capecitabine (GEMCAP group), and the other group received a single chemotherapy regimen of GEM (GEM group). The results showed that the OS of GEMCAP group was slightly prolonged, but the difference was not statistically significant. The 1-year overall progression free survival (PFS) in GEMCAP group was significantly higher than that in GEM group (13.9% vs 8.4%, P = 0.004). Herrmann et al. (51) showed that there was no significant difference in mOS and 1-year survival between GEMCAP group and gem group, but efficacy analysis showed that patients with higher KPS had longer mOS, and GEMCAP regimen could significantly improve PFS (P = 0.022). The National Comprehensive Cancer Network (NCCN) has classified the GEMCAP protocol as an alternative for advanced pancreatic cancer treatment, and shows that the premise of choosing this protocol bring better physical fitness and behavioral status (KPS:90-100 score).

Japan proposed GEM plus S-1 as a chemotherapy regimen for advanced pancreatic cancer. Okabayashi (52) and other studies suggested that S-1 and GEM alone had no significant difference in OS. However, Meta-analysis of Li (53) in patients with pancreatic cancer after S-1 combined with GEM adjuvant therapy showed that GEM and S-1 in patients with non resectable pancreatic cancer significantly improved the patient’s OS and PFS. Wada et al. (54) Proposed GEM combined with S-1 chemotherapy twice a week, which can reduce adverse reactions and economic burden without weaken therapeutic efficacy.

Heinemann and Colucci (55) and other phase III clinical trials confirmed that GEM combined with platinum chemotherapy drugs did not improve the survival time of patients with Heinemann compared with GEM chemotherapy alone. A total of 400 patients with advanced pancreatic cancer were randomized to receive GEM plus cisplatin or GEM monotherapy. The results showed that there was no significant difference in mOS and PFS between the two groups. However, the results of a large meta-analysis showed that GEM combined with cisplatin could effectively improve the quality of life of patients compared with GEM monotherapy group (P = 0.010). Therefore, NCCN lists GEM combined platinum chemotherapy drugs as one of the treatment options for advanced pancreatic cancer, but limited to patients with familial pancreatic cancer.

A series of phase I clinical trials confirmed that GEM combined with oxaliplatin, irinotecan or pemetrexed cannot significantly prolong OS in patients with pancreatic cancer (47). GERCOR and GISCAD tests showed that GEM combined with oxaliplatin can improve PFS, but it has no significance on OS (56).

#### 3.2.4 Chemotherapy for pancreatic cancer patients with BRCA gene mutation

Although GEMCAP combined with cisplatin is not widely recommended in the clinical treatment of early pancreatic cancer, studies have confirmed that familial pancreatic cancer or pancreatic cancer with BRCA mutation is more sensitive to platinum-based chemotherapy (57).

BRCA1 and BRCA2 mutations can lead to ineffective repair of damaged DNA in homologous recombination and increase the risk of malignant tumor. Cisplatin, as an alkylating drug, can combine with DNA to form intrastrain crosslinks, change the structure of DNA and affect DNA replication. Under normal circumstances, these crosslinks can be repaired by homologous recombination, but patients with BRCA gene mutation cannot complete effective repair, BRCA deficient cells are more sensitive to platinum-based chemotherapy. In a retrospective study conducted by Johns Hopkins University in 2010, 468 patients with metastatic pancreatic cancer who were treated with cisplatin-based chemotherapy were evaluated. It was found that patients with family history of breast cancer, ovarian cancer or pancreatic cancer had significantly longer mOS than those without such family history (22.9mo vs 6.3 mo). P<0.01). At the same time, Lowery (58) and other research results also showed that BRCA1 or BRCA2 mutant pancreatic cancer patients can use PARP inhibitor or platinum chemotherapy drug to achieve 27.6 months on mOS. PARP family protein binding with DNA and participate in the repair of DNA damage. Therefore, inhibition of PARP can hinder the damage and repair of DNA and ultimately induce cell apoptosis (59). These two studies all suggest that platinum-based chemotherapy drugs may be effective in improving mOS in familial pancreatic cancer or BRCA gene mutation patients.

#### 3.2.5 Oxaliplatin + folic acid + fluorouracil regimen

CONKO-003 trial of second-line chemotherapy for pancreatic cancer showed that compared with folate + fluorouracil (FF) regimen, the OFF regimen increased relative to GEMCAP resistant patients (2.9 mo vs 2.0 mo, P=0.019), OS was also significantly prolonged (5.9 mo vs3.3mo, P=0.01), but the neurotoxicity of the regimen was apparently higher than that of the regimen (60). The NCCN guidelines recommend OFF regimen as one of second-line chemotherapy regimens for GEMCAP resistance in advanced pancreatic cancer.

#### 3.2.6 5-Fu + folic acid + irinotecan + oxaliplatin regimen

In the ACCORD II/III trial, 342 patients with metastatic pancreatic cancer who had not received any treatment were randomized to receive FOLFIRINOX chemotherapy or GEMCAP monotherapy. The former mOS (11.1 mo vs 6.8 mo, P<0.001) or PFS (6.4 mo vs 3.3 mo, P<0.001) are significantly higher than the latter, and the tumor is more sensitive to the former regimen (31.6% vs 9.4%, P<0.001), which suggests that combined chemotherapy can improve the survival rate of metastatic pancreatic cancer patients compared with single dose of chemotherapy (61). Compared with GEMCAP monotherapy, FOLFIRINOX regimen had a higher incidence of grade 3 and 4 adverse reactions, but the 6 months health status and quality of life scores showed that the overall quality of life of FOLFIRINOX group was higher than that of GEMCAP group, which may be related to the significantly improved survival rate of FOLFIRINOX regimen (62). Currently, the FOLFIRINOX regimen is considered to be a first-line chemotherapy regimen of advanced pancreatic cancer in general condition. The combination of 27 GEMCAP and paclitaxel regimen is rich in stroma, which can block chemotherapeutic drugs from entering cancer cells and increase chemotherapy resistance. In recent years, a new scheme of paclitaxel combined with GEMCAP for metastatic pancreatic cancer has been proposed abroad. Nano paclitaxel is a combination of human albumin and paclitaxel by using nanotechnology to import drugs into cancer cells in the form of nanoparticles and increase the bioavailability of drugs. The uptake of paclitaxel nanoparticles by pancreatic stromal cells requires specific albumin binding proteins, such as cysteine rich secreted protein (SPARC). In a phase I/II clinical trial, the expression level of SPARC in 36 patients was detected by immunohistochemistry and used as a biomarker, the patients were divided into high expression SPARC group and low expression SPARC group. The results showed that the mOS of high expression SPARC group was significantly higher than that of low expression SPARC group, which suggested that GEMCAP combined with Nano-paclitaxel showed important antitumor activity. However, another phase II trial using paclitaxel as a second-line treatment for metastatic pancreatic cancer has found no significant correlation between the expression of SPARC and prognosis. In phase III clinical trials such as Von Hoff, a total of 861 patients with untreated advanced pancreatic cancer were randomly divided into GEMCAP combined with paclitaxel chemotherapy or GEMCAP single chemotherapy. The results showed that GEMCAP, combined with paclitaxel group had significant improvement in mOS, PFS and tumor sensitivity, but the incidence of myelosuppression and peripheral neuritis in this group was equally higher. MPACT detailed analysis of SPARC expression and patient survival at the 2014 European Society of Clinical Oncology Conference also showed that SPARC was not associated with patient survival.

Currently, GEMCAP combined with paclitaxel or FOLFIRINOX is a first-line treatment for pancreatic cancer. However, pancreatic cancer is a highly malignant tumor, and nearly half of the patients are ineffective for first-line treatment. At this time, chemotherapy drugs such as fluorouracil, capecitabine, pemetrexed and oxaliplatin can play an essential role. However, there is no standardized treatment plan for patients with advanced pancreatic cancer who are tolerant of first-line and second-line chemotherapy.

#### 3.2.7 Neoadjuvant chemotherapy for the operation of pancreatic cancer

For the resectable or borderline resectable pancreatic cancer patients, they can receive the neoadjuvant chemotherapy or adjuvant therapy (63, 64). There were many clinical trials suggested that the FOLFIRINOX add radiotherapy is the preferred new adjuvant therapy (41, 65, 66). Janssen QP, et al. reported that 351 patients (68.6%) were treated with FOLFIRINOX alone (8 studies) and 161 patients (31.4%) were treated with FOLFIRINOX and radiotherapy (7 studies). The pooled estimated median OS was 21.6 months (range 18.4–34.0 mo) for FOLFIRINOX alone and 22.4 months (range 11.0–37.7 mo) for FOLFIRINOX with radiotherapy. The pooled resection rate was similar (71.9% vs. 63.1%, p = 0.43) and the pooled R0 resection rate was higher for FOLFIRINOX with radiotherapy (88.0% vs. 97.6%, p = 0.045). Other pathological outcomes (ypN0, pathologic complete response, perineural invasion) were comparable (67). Giovinazzo F, et al. (68)found that gemcitabine based neo-adjuvant therapies (GEM-NAT) in borderline resectable pancreatic ductal adenocarcinoma (BR-PDAC). A meta-analysis of individual participant data (IPD) was conducted on 271 patients who received GEM-NAT. Pooled median patient-level OS was 22.2 months (95%CI 19.1–25.2). R0 rates ranged between 81 and 95% (I2 = 0%, p = 0.64), respectively. Median OS was 27.8 months (95%CI 23.9–31.6) in the patients who received NAT-GEM followed by resection compared to 15.4 months (95%CI 12.3–18.4) for NAT-GEM without resection and 13.0 months (95%CI 7.4–18.5) in the group of patients who received upfront surgery (p < 0.0001). R0 rates ranged between 81 and 95% (I2 = 0%, p = 0.64), respectively. Overall survival in the R0 group was 29.3 months (95% CI 24.3–34.2) vs. 16.2 months (95% CI 7·9–24.5) in the R1 group (p = 0·001). GEM-NAT may result in a good palliative option in non-resected patients because of progressive disease after neoadjuvant treatment (68).

The standard treatment of resectable pancreatic cancer is surgery followed by adjuvant chemotherapy. Neoadjuvant chemotherapy appears to be equally efficient in converting irresectable in resectable disease and more efficient with regard to systemic tumor progression and overall survival compared to neoadjuvant chemoradiation therapy. Despite these convincing findings from mostly small phase II trials, neoadjuvant therapy has not yet proven superiority over upfront surgery in randomized trials (63, 66, 69–72). Vivarelli et al (64) suggested that the choice of the best multimodal treatment of resectable pancreatic cancer should probably be based on the biological behavior of the tumor rather than on the loco-regional staging of the tumor, which currently represents the cornerstone of the decision-making process with regard to first-line treatment. More effective and individualized systemic therapeutic regimens will probably stem from a better knowledge of clinic-pathological prognostic factors such as molecular profiling and novel biomarkers.

### 3.3 Radiotherapy

Radiotherapy is an important treatment for pancreatic cancer, which is the first choice for locally advanced pancreatic cancer (73). Generally speaking, the sensitivity of pancreatic cancer to radiotherapy alone is rather poor. The current view is that radiotherapy can be combined on the basis of chemotherapy for patients with advanced stage, but there are still differences in the effectiveness. A study has shown that chemoradiotherapy improves overall survival compared with chemotherapy alone, but the adverse reactions are also significantly enhanced. Another study suggested that the overall survival rate after chemoradiotherapy was slightly lower than that after chemotherapy alone (15.3 mo vs 16.5 mo). In last years, the radiotherapy technology has also been improved significantly, such as three-dimensional conformal radiotherapy, which focuses on raising the radiation dose and gradually improving the stereotactic radiotherapy technology of primary tumor. Although there are many problems with these technologies, the latest radiotherapy combined with chemotherapy is very promising for the treatment of patients with advanced pancreatic cancer.

### 3.4 Targeted therapy

Epidermal growth factor receptor (EGFR) is a transmembrane tyrosine kinase receptor that plays an important role in cell cycle regulation. 90% of all pancreatic cancer samples are highly expressed in EGFR. Therefore, targeting small molecule inhibitors of EGFR tyrosine kinase domain is a promising drug for cancer therapy. In a large clinical phase II trial, 569 patients with advanced pancreatic cancer were randomly divided into GEM combined with erlotinib or GEM monotherapy. The results showed that mOS and PFS in the combination group were obviously higher than those in the single drug group. Subsequently, the trial also analyzed the number of KRAS and EGFR in 117 patients, and found that neither of them could predict the longer survival of patients with combination regimen. In addition, EGFR monoclonal antibody (cetuximab) combined with GEM was also used. Immunohistochemistry showed that 92% of the tumor tissues were EGFR positive, but it did not improve the mOS, PFS or tumor sensitivity. Türeci Ö found that zolbetuximab-induced antibody-dependent cell-mediated cytotoxicity (ADCC), and in mouse xenograft tumors derived from human pancreatic cancer cell lines, including GEM-refractory ones, zolbetuximab slowed tumor growth, benefited survival, and attenuated metastases development (74).

With the research of pancreatic cancer related genes and signaling pathways, targeted therapy has become a new method for the treatment of pancreatic cancer, including directly targeting tumor antigen, growth factor receptor, changing gene or biochemical channels, directly responding to host immune response (75). Olaparib can be used for targeted therapy in pancreatic cancer patients with BRCA1/2 mutation (76).

Activation of the tyrosine kinase domain of EGFR to activate the downstream RAS/RAF/MEKPI3K/AKT and JAK/STAT signaling pathways is essential for cell proliferation and survival. This makes the research and development of EGFR small molecule inhibitors become a hot spot in the field of tumor therapy. Currently, EGFR inhibitors such as Nimotuzumab and Afatinib are currently undergoing phase I clinical trials. In addition, insulin-like growth factor receptor (IGFR) can also regulate cell proliferation by activating signal pathways such as PI3K/AKT, but IGFR monoclonal antibodies and MK-0646 have not been effective for pancreatic cancer.

On the other hand, 90% of pancreatic cancer has a mutation in the KRAS gene, which activates RAF/MEK/ERK and PI3K/AKT channels, leading to uncontrollable cell growth. This makes KRAS a potential target for pancreatic cancer treatment. However, its inhibitors, either alone or in combination, are not effective in the treatment of pancreatic cancer. Therefore, the inhibitors of its downstream signaling pathway are tried to treat pancreatic cancer, such as the use of MEK1/2, an inhibitor of the oral administration of the drug. But compared with GEM, the drug does not prolong the mOS of patients with pancreatic cancer. Trametinib is a reversible MEK1/2 inhibitor. Although it has not significantly improved the mOS of patients, it has been used in the treatment of advanced pancreatic cancer. At present, more drugs blocking KRAS signaling pathway are being developed, among which PI3K inhibitors and AKT inhibitors have entered the clinical trial stage.

### 3.5 Immunotherapy

Programmed death 1(PD-1)/programmed cell death-Ligand 1(PD-L1) immunotherapy can be considered for pancreatic cancer patients with disease progression after surgery or first-line chemotherapy (47, 77, 78). MSI or MMR genes closely related to pancreatic cancer should be detected before immunotherapy (7, 79, 80). Immunotherapy with antibodies targeting PD-1, PD-L1, cytotoxic T lymphocyte associated antigen 4 (CTLA-4) has not shown clinical activity in unselected pancreatic cancer, emphasizing the need for combination of immunotherapy approaches or other therapeutic strategies (81).

Pancreatic cancer cells are able to escape human immune system monitoring by various mechanisms, such as negative regulation of T cell response (82), secretion of cytokines inhibiting the immune system, and down regulation of major histocompatibility complex-I (MHC-I) expression. This provides a basis for the discovery of tumor specific antigen, the development of tumor vaccine and antibody (83).

Ipilimumab is a specific monoclonal antibody against CTLA-4 (84). Its combination with CTLA-4 can enhance the activity and function of T cells. It has been confirmed by FDA for the treatment of melanoma. Currently, clinical trials have combined it with the FOLFIRINOX scheme and allogeneic tumor vaccine in the treatment of pancreatic cancer. Tumor vaccine is promising in the field of tumor immunotherapy. Allogeneic pancreatic cancer vaccine is injected into another patient from a cancer cell vaccine. It hopes to express specific tumor antigens and be recognized by the host immune system, thereby stimulating the immune response to the host’s own tumor. The only tumor vaccine approved by FDA is the Sipuleucel-T cancer vaccine, which is used to treat steroid resistant prostate cancer. CRS-207 is still undergoing the studying. It is an attenuated vaccine of Lester, which can express mesothelin (mesothelin is a glycoprotein overexpressed on pancreatic cancer cell surface), and its mechanism is bacteria invading macrophages to produce mesothelin. Subsequently, activation of mesothelin cytotoxic T cells eventually induces apoptosis of tumor cells expressing mesothelin. Currently, phase CRS-207 clinical trials of CRS-207 and GVAX, a master cell vaccine expressing human granulocyte macrophage colony-stimulating factor, are being carried out. Jung and his colleges found that the combination of Navoximod and atezolizumab demonstrated acceptable safety, tolerability, and pharmacokinetics for patients with advanced cancer (NCT02471846) (85).

Other immunotherapy (80, 86–90), such as tumor antibody development and transformation of lymphocytes, are promising new technologies for the treatment of pancreatic cancer. However, more clinical data are needed to confirm the clinical value. CDK1/2/5 inhibition by dinaciclib provides a novel strategy to overcome IFNG-triggered acquired resistance in pancreatic tumor immunity (91).

## 4 Necessity of MDT

The condition of patients with pancreatic cancer is complex. At present, the treatment of pancreatic cancer in large hospitals in China involves pancreatic surgery, gastroenterology, oncology, radiotherapy, pathology, medical imaging, nuclear medicine and other clinical fields. Each department has certain limitations. Therefore, MDT should go through the whole process of pancreatic cancer treatment, including the choice of treatment decision, surgery and chemoradiotherapy, and targeting (39, 92, 93). It is of great significance for the treatment of patients with pancreatic cancer to combine various departments to achieve the best therapeutic effect.

**Figure 1:**
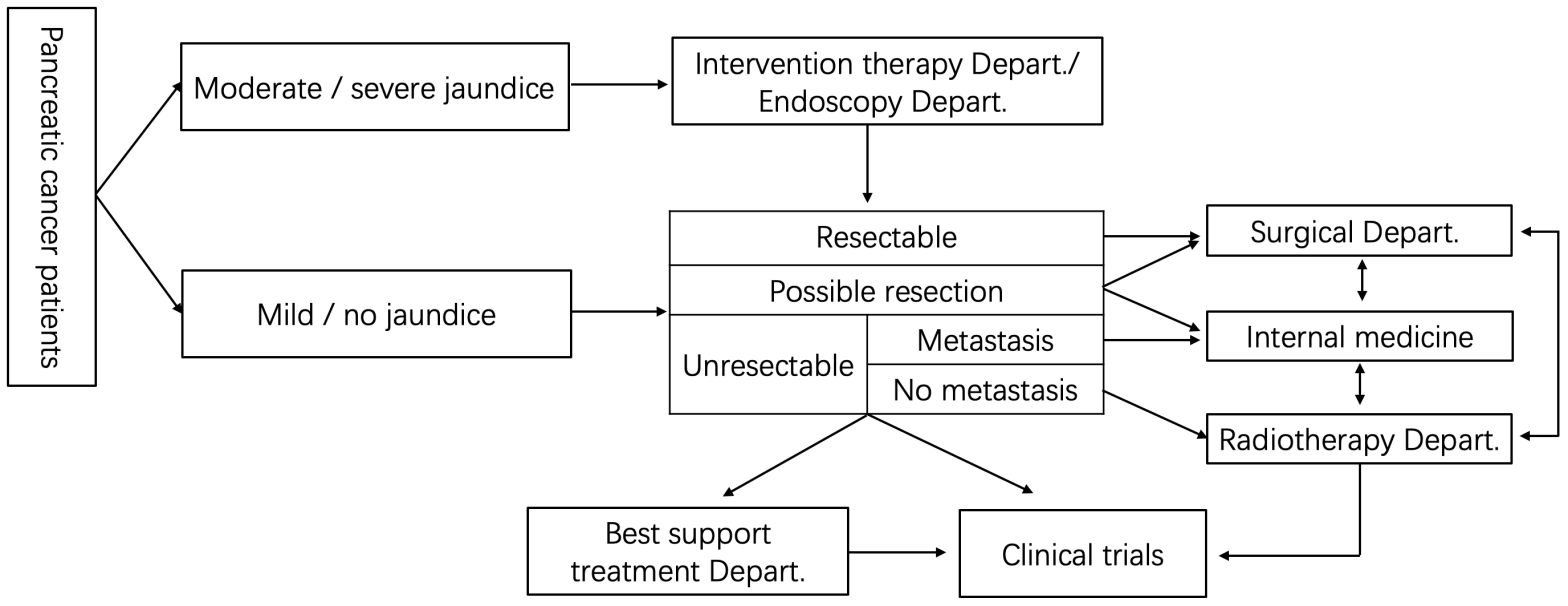
The path map of MDT model in pancreatic cancer.

In recent years, MDT model has become one of the important models of international medicine (94–96). Its purpose is to transform the traditional individual and empirical medical model into a modern group cooperative decision-making model. The National Comprehensive Cancer Network (NCCN) guidelines bring MDT discussion into the necessary procedures, and the Chinese Medical Association has also brought MDT into the treatment of each patient with pancreatic cancer (97, 98), including medicine, technology, nursing and other disciplines, the use of multidisciplinary linkage can improve the survival of patients and ensure the quality of life of patients. And the path map of MDT model in pancreatic cancer as show **Figure 1** (97, 98). MDT treatment mode brings together the advantages of various departments, and plays an irreplaceable role in improving the treatment level, formulating the corresponding treatment plan, reducing over treatment, and diagnosis and treatment of pancreatic cancer in China (99).

## 5 Current landscape of MDT

At present, there are still a few doctors in MDT who lack the awareness of multidisciplinary diagnosis and treatment. Due to the limitations of the existing medical system and the different treatment methods of pancreatic cancer belong to different disciplines. It is easy for some patients with pancreatic cancer not to get the most reasonable treatment or to receive a single treatment repeatedly in a single specialty for a long time.

The MDT of pancreatic cancer regularly holds MDT forums to discuss difficult cases, improve the level of diagnosis and treatment, and formulate personalized and optimal treatment plan for patients in strict accordance with the corresponding clinical treatment guidelines. The operation and treatment process of MDT team for pancreatic cancer follow NCCN treatment guidelines and Chinese pancreatic cancer treatment guidelines. Although the working process of MDT is perfect, some doctors can’t participate in it for some reasons, which leads to the interruption of MDT and can’t implement it well. The most challenge when conduct MDT model maybe how to make the best choice in the face of multiple treatment decisions. Usually, the surgery department should act as the leader in MDT model, and when disagreement happens, the pancreatic surgeon makes the decision.

At present, there are some limitations in the implementation of MDT, such as nutritionists and psychiatrists cannot play a role in the whole treatment of patients, so the benefits of MDT for patients will be impaired.

Although MDT of pancreatic cancer is mostly difficult cases, it would promote the communication between domestic and foreign counterparts, but in the actual process, there is not enough communication at home and abroad (100, 101). MDT discuss the diagnosis and treatment of a case in various disciplines, which is a good opportunity for young doctors to learn and improve, and is conducive to the cultivation of young doctors’ diagnosis and treatment thinking. But in fact, young doctors rarely participate in MDT due to busy work and other reasons, which is not conducive to talent cultivation and talent echelon construction.

## 6 Future prospects of MDT

In the implementation of MDT, there should be a distribution mechanism to protect the income and rights of doctors and show respect for doctors’ work, which can improve the enthusiasm of doctors in MDT and ensure the continuous operation of MDT.

Although MDT model runs through the whole process of diagnosis and treatment of pancreatic cancer, which can fully integrate the resources of various disciplines, give full play to the advantages of disciplines, and seek individualized diagnosis and treatment scheme for patients, how to break through the bottleneck of diagnosis and treatment of pancreatic cancer still depends on the progress of science and technology to improve the proportion of early diagnosis of pancreatic cancer. At the same time, the research on the treatment of pancreatic cancer still cannot stop, hoping to explore a more valuable treatment. With the help of MDT, patients will benefit more, especially those conditions with poor therapeutic effect, such as pancreatic cancer. As for how to choose a variety of treatment methods in the future, the expand of MDT still needs to think carefully.

We can try the Internet + MDT (e MDT) model for pancreatic cancer (102, 103). E-MDT should be based on the current perfect MDT model, combined with Internet, 5th-Generation (5G), Artificial Intelligence (AI) Technology and big data to build an internet medical consortium cloud platform integrating medical record data collection, imaging, laboratory, pathology, remote consultation, surgical demonstration and remote learning, providing remote consultation, joint outpatient service, mobile ward round, teaching and training and other remote services; Integrating convenient mobile medicine, the cloud platform will become a telemedicine platform that can support multi person, multi terminal (personal computer (PC), mobile phone, iPad, etc.) integration and multi scene applications; it can be moved forward to the consulting room, patients’ bed, mobile phone terminal for online consultation, multi person multidisciplinary consultation and mobile consultation at any time, which will facilitate the development of consultation business between different medical institutions.

## Author contributions

QS and WY conceived the project. All authors collected and analyzed the data. QS and XC prepared the figure. QS wrote the manuscript. All authors contributed to the article and approved the submitted version.
